# Antineutrophil cytoplasmic antibodies in patients treated with methimazole: a prospective Brazilian study^☆^

**DOI:** 10.1016/j.bjorl.2018.05.014

**Published:** 2018-07-17

**Authors:** Gabriela Costa Andrade, Flavia Coimbra Pontes Maia, Gabriela Franco Mourão, Pedro Weslley Rosario, Maria Regina Calsolari

**Affiliations:** Programa de Pós-graduação e Serviço de Endocrinologia, Instituto de Ensino e Pesquisa da Santa Casa de Belo Horizonte, Belo Horizonte, MG, Brazil

**Keywords:** Antineutrophil cytoplasmic antibodies, Methimazole, Vasculitis, ANCA, Metimazol, Vasculite

## Abstract

**Introduction:**

The side effects of antithyroid drugs are well known. Antineutrophil cytoplasmic antibody-associated vasculitis is a severe adverse reaction. Most studies evaluating antineutrophil cytoplasmic antibodies related to antithyroid drugs have been carried out with patients treated with propylthiouracil, but less information is available for methimazole. Furthermore, most studies that investigated antineutrophil cytoplasmic antibodies related to antithyroid drugs were conducted on Asian populations.

**Objective:**

To evaluate the frequency of antineutrophil cytoplasmic antibodies and antineutrophil cytoplasmic antibodies-positive vasculitis in an adult population of Brazilian patients treated with methimazole.

**Methods:**

This was a prospective study. We evaluated patients ≥18 years with Graves’ disease who have been using methimazole for at least 6 months (Group A, *n* = 36); with Grave's disease who had been previously treated with methimazole but no longer used this medication for at least 6 months (Group B, *n* = 33), and with nodular disease who have been using methimazole for at least 6 months (Group C, *n* = 13).

**Results:**

ANCA were detected in 17 patients (20.7%). Four patients (4.9%) had a strong antineutrophil cytoplasmic antibodies-positive test. The frequency of antineutrophil cytoplasmic antibodies was similar in the groups. When Groups A and B were pooled and compared to Group C to evaluate the influence of Grave's disease, and when Groups A and C were pooled and compared to Group B to evaluate the influence of methimazole discontinuation, no difference was found in the frequency of antineutrophil cytoplasmic antibodies. No difference was observed in sex, age, etiology of hyperthyroidism, anti-TSH receptor antibodies, dose or time of methimazole use between patients with versus without antineutrophil cytoplasmic antibodies. The titers of these antibodies were not correlated with the dose or time of methimazole use. None of the antineutrophil cytoplasmic antibodies-positive patient had clinical event that could potentially result from vasculitis.

**Conclusion:**

This clinical study of a Brazilian population shows a considerable frequency of antineutrophil cytoplasmic antibodies in patients treated with methimazole but the clinical repercussion of these findings remains undefined.

## Resumo

**Introdução:**

Os efeitos adversos de drogas antitireoidianas são conhecidos. Vasculite associada a anticorpos anticitoplasma de neutrófilos é uma reação adversa grave. A maioria dos estudos que avaliam anticorpos anticitoplasma de neutrófilos relacionado a drogas antitireoidianas envolveu pacientes tratados com propiltiouracil, entretanto menos informação se encontra disponível para o metimazol. Além disso, a maioria dos estudos que investigaram anticorpos anticitoplasma de neutrófilos relacionado a drogas antitireoidianas foi conduzida em populações asiáticas.

**Objetivo:**

Avaliar a frequência de anticorpos anticitoplasma de neutrófilos e vasculite anticorpos anticitoplasma de neutrófilos-positivo em uma população adulta de pacientes brasileiros tratados com metimazol.

**Método:**

Este foi um estudo prospectivo. Avaliamos pacientes ≥ 18 anos com doença de Graves com o uso de metimazol há pelo menos seis meses (Grupo A, n = 36); com doença de Graves previamente tratados com metimazol, mas que não usaram esse medicamento por pelo menos seis meses (Grupo B, n = 33) e com doença nodular em uso de metimazol há pelo menos seis meses (Grupo C, n = 13).

**Resultado:**

Anticorpos anticitoplasma de neutrófilos foram detectados em 17 pacientes (20,7%). Quatro pacientes (4,9%) tinham anticorpos anticitoplasma de neutrófilos fortemente positivos. A frequência de anticorpos anticitoplasma de neutrófilos foi semelhante nos grupos. Quando os Grupos A e B foram somados e comparados ao Grupo C para avaliar a influência da doença de Graves, e quando os Grupos A e C foram somados e comparados ao Grupo B para avaliar a influência da interrupção do metimazol, não foi encontrada diferença na frequência de anticorpos anticitoplasma de neutrófilos. Não houve diferença em relação a sexo, idade, etiologia do hipertireoidismo, anticorpos antirreceptor de TSH, dose ou tempo de uso de metimazol entre pacientes com e sem anticorpos anticitoplasma de neutrófilos. Os títulos desses anticorpos não se correlacionaram com dose ou tempo de uso de metimazol. Nenhum paciente anticorpos anticitoplasma de neutrófilos-positivo apresentou evento clínico resultante de vasculite.

**Conclusão:**

Este estudo clínico de uma população brasileira apresenta frequência considerável de anticorpos anticitoplasma de neutrófilos em pacientes tratados com metimazol, mas a repercussão clínica desse achado permanece indefinida.

## Introduction

Antithyroid drugs (ATD) are widely prescribed for the treatment of hyperthyroidism. In many patients with Graves’ disease these drugs are initiated and subsequently discontinued, with persistence of euthyroidism, while in cases in which the maintenance of euthyroidism is not possible after drug discontinuation the patient can be treated definitively by surgery or radioiodine or can be maintained on a long-term ATD therapy.^1^, ^2^, ^3^, ^4^, ^5^ Different from a few years ago, the last possibility has gained increasing acceptance.^2^, ^3^, ^4^, ^5^

The side effects of ATD are well known. Like hepatotoxicity and agranulocytosis, antineutrophil cytoplasmic antibody(ANCA)-associated vasculitis is a severe adverse reaction.^1^, ^2^, ^6^ In contrast to the other adverse effects that usually occur in the first months of ATD use, the frequency of ANCA-positive vasculitis seems to increase with the duration of therapy.^2^, ^6^ Thus, in patients who do not exhibit adverse drug reactions in the first months of treatment, concern over side effects is lower, except for ANCA-positive vasculitis.

Most ANCA-positive patients do not have clinical vasculitis.^2^, ^6^ However, since the presence of these antibodies is the condition necessary for the clinical event, knowledge about the relationship between ATD use and an ANCA-positive test is important. Most studies evaluating ANCA related to ATD involved patients treated with propylthiouracil (PTU), while less information is available for methimazole (MMI).^2^, ^6^ It should be noted that MMI is the ATD of choice, except for the first trimester of gestation.^1^, ^2^ Furthermore, most studies that investigated ANCA and/or ANCA-positive vasculitis related to ATD were conducted on Asian populations and information for American populations is therefore sparse.^2^, ^6^ The latest and largest review on this topic^6^ identified only three studies that evaluated the frequency of ANCA in non-Asian patients treated with MMI and none of them was conducted in the United States.^6^ Our literature search found no clinical trial with these features that was published after this review.

For these reasons, the objective of this prospective clinical study was to evaluate the frequency of ANCA and ANCA-positive vasculitis in an adult population of Brazilian patients treated with MMI.

## Methods

This was a prospective study. The study was approved by the local Research Ethics Committee (CAAE 72093917.6.0000.5138/no.2.194.997) and the subjects gave written informed consent.

Patients of both sexes ≥18 years consecutively seen between June and October 2017 at the thyroid outpatient clinic of the Endocrinology Service of Santa Casa de Belo Horizonte were initially selected. The patients were included in the study when they met one of the following criteria: (i) Diagnosis of Graves’ disease and use of MMI for at least 6 months (Group A); (ii) Diagnosis of Graves’ disease in remission and previous treatment with MMI but no use of this medication for at least 6 months (Group B), or (iii) Diagnosis of toxic nodular disease and use of MMI for at least 6 months (Group C). The diagnosis of hyperthyroidism was made when TSH concentrations were persistently <0.1 mIU/L and free T4 and/or T3 were elevated. The etiology was defined based on the presence of ophthalmopathy, circulating anti-TSH Receptor Antibodies (TRAb) and imaging methods (ultrasonography and scintigraphy) revealing goiter with diffuse hyperuptake or nodule(s) with focal hyperuptake and suppression of remnant tissue. Patients exposed to other drugs also associated with an ANCA-positive result, such as hydralazine, allopurinol, sulfasalazine, d-penicillamine, minocycline and methotrexate, were excluded.

All patients were submitted to the measurement of ANCA and investigation of clinical events that could potentially result from vasculitis (systemic, cutaneous, respiratory, renal and neurological conditions)^6^ and that occurred between the beginning of treatment with MMI and evaluation in this study. The patients were in euthyroid state at the time when ANCA tests were performed.

Following international recommendations, screening for ANCA was performed by indirect immunofluorescence because of its high sensitivity.^6^, ^7^ When positive (considering positivity titers at or above 1/20), this method reveals two patterns of fluorescence: cytoplasmic (c-ANCA) or perinuclear (p-ANCA). Titers at or above 1/80 were considered strong positive.^8^

Fisher's exact test and the Chi-squared test were used for statistical analysis (comparison between the groups). A *p*-value less than 0.05 was considered significant.

## Results

The characteristics of the patients are shown in Table 1.

**Table 1:** Characteristics of patients and frequency of ANCA in the groups studied.

	Group A (*n* = 36)	Group B (*n* = 33)	Group C (*n* = 13)
Sex, Female (F)/Male (M)	24 F/12 M	27 F/6 M	11 F/2 M
Age (mean, years)	52	54	74
MMI dose (mean, mg/day)	14	16^a^	10
Time of MMI use (mean, months)	60	84^a^	60
Time without MMI (mean, months)	–	36	–
TRAb-positive	20 (55.5%)	17 (51.5%)	0
ANCA-positive test^e^	8 (22.2%)^b^	8 (24.2%)^c^	1 (7.7%)^d^
Strong ANCA-positive test^f^	1 (2.7%)	2 (6%)	1 (7.7%)

Group A, Graves’ disease using MMI for at least 6 months; Group B, Graves’ disease without MMI for at least 6 months; Group C, toxic nodular disease using MMI for at least 6 months.

ANCA, antineutrophil cytoplasmic antibodies; TRAb, anti-TSH receptor antibodies; MMI, methimazole.

a Considering the time when patients were treated with MMI.

b ANCA-positive detected after 6, 14, 22, 24, 36, 38, 74 and 144 months using MMI, respectively.

c These patients were treated with MMI for 10, 19, 27, 36, 36, 41, 48 and 51 months, respectively.

d ANCA-positive detected after 36 months using MMI.

e Group A vs. B (*p* = 0.5); Group A vs. C (*p* = 0.4); Group B vs. C (*p* = 0.4).

f Group A vs. B (*p* = 0.6); Group A vs. C (*p* = 0.5); Group B vs. C (*p* = 1.0).

ANCA were detected in 17 patients (20.7%), including p-ANCA in 15 and c-ANCA in 2. Four patients (4.9%) had a strong ANCA-positive test. The frequency of ANCA was similar in the three groups (Table 1). When Groups A and B were pooled and compared to Group C to evaluate the influence of Graves’ disease on the frequency of ANCA, an apparent difference was observed but this difference did not reach statistical significance (*p* = 0.28). Furthermore, when Groups A and C were pooled and compared to Group B to evaluate the influence of MMI discontinuation on the frequency of ANCA, no difference was found (*p* = 0.58).

The patients with ANCA were compared to those without ANCA and no difference was observed in sex, age, etiology of hyperthyroidism, presence of TRAb, dose or time of MMI use (Table 2). In patients with ANCA, the titers of these antibodies were not correlated with the dose or time of MMI use.

**Table 2:** Comparison of patients with versus without ANCA.

	ANCA-positive (*n* = 17)	ANCA-negative (*n* = 65)	*p*-Value
Sex, Female (F)/Male (M)	15 F/2 M	47 F/18 M	0.2
Age (mean, years)	59.4	55.2	0.4
Etiology of hyperthyroidism, Graves’ Disease (GD)/Nodular Disease (ND)	16 GD/1 ND	53 GD/12 ND	0.3
TRAb-positive	10 (58.8%)	27 (41.5%)	0.3
MMI dose (mean, mg/day)^a^	12	15	0.7
Time of MMI use (mean, months)^a^	60	72	0.9

ANCA, antineutrophil cytoplasmic antibodies; TRAb, anti-TSH receptor antibodies; MMI, methimazole.

a Considering the time when patients who no longer used MMI were treated.

Finally, none of the 17 ANCA-positive patients exposed to MMI had clinical event that could potentially result from vasculitis. Transient cutaneous symptoms not attributable to vasculitis were observed in 5 patients (6%), with no difference between those with and without ANCA (*p* = 0.35).

## Discussion

Some characteristics of the present study deserve to be highlighted. The patients included in the present study were exposed to MMI. MMI is the drug of choice for the treatment of hyperthyroidism, except for the first trimester of gestation.^1^, ^2^ In addition, in contrast to PTU, the association of MMI with an ANCA-positive test is much less studied.^2^, ^6^ Although case reports exist, there are few clinical studies evaluating the frequency of ANCA in patients treated with MMI and an even smaller number involve non-Asian populations,^2^, ^6^ none of them was conducted in the United States. In addition to being the first report investigating this occurrence in a Brazilian population, our study was prospective and included consecutive patients, a fact minimizing the possibility of under- or overestimation of the frequency of ANCA. The evaluation of three groups exposed to MMI also permitted to explore the influence of Graves’ disease and of drug discontinuation on an ANCA-positive test.

The frequency of ANCA in our patients exposed to MMI was 20%. Previous studies reported variable results of an ANCA-positive test in patients treated with MMI: 0%,^9^, ^10^, ^11^ 3.5%,^12^ 7%,^13^, ^14^, ^15^, ^16^ and 16%.^17^ Although the frequency found by us was not low, it is important to recognize that we did not evaluate patients not exposed to MMI for comparison. Six previous studies evaluating ANCA in patients treated with MMI included a control group not treated with ATD.^9^, ^10^, ^11^, ^13^, ^16^, ^17^ In these studies, ANCA was uncommon in patients not exposed to ATD, with a frequency ranging from 0% to 6%. In addition, specifically in the adult Brazilian population, the frequency of ANCA in adults without autoimmune disease was 0%.^18^, ^19^ In patients treated only with MMI (without PTU), the frequency of ANCA was also low in five of the six studies, ranging from 0% to 9%.^9^, ^10^, ^11^, ^13^, ^16^ However, a larger study reported a higher frequency of ANCA in patients treated with MMI (15.9% vs. 3.8%).^17^ When the results of these studies were compiled,^9^, ^10^, ^11^, ^13^, ^16^, ^17^ ANCA were detected in 3/161(1.9%) patients not exposed to ATD versus 44/405 treated with MMI (10.9%) (*p* = 0.0002). We also found ANCA in one patient with toxic nodular disease, suggesting that a positive antibody test related to the use of MMI does not only occur in patients with autoimmune thyroid disease (Graves’). Only two previous clinical studies that evaluated ANCA in patients treated with MMI included nodular disease^13^, ^15^ and one of them also reported a patient with multinodular goiter and an ANCA-positive test.^13^ Finally, despite the homology between Thyroperoxidase (TPO) and Myeloperoxidase (MPO), a p-ANCA-positive test (whose main antigen is MPO) is not due to cross-reactivity with TPOAb.^17^

In the present study, none of the patients with ANCA had clinical vasculitis. We recognize that long-term follow-up is necessary to ensure that the nine patients with ANCA who were maintained on MMI will not have manifest disease. In the series of Harper et al.,^17^ a study involving the largest number of ANCA-positive patients treated with MMI, none of the 40 participants had clinical vasculitis. These data suggest that an ANCA-positive test in patients treated with MMI is unlikely to result in clinical repercussions. The rarity of vasculitis in patients maintained on a low-dose MMI for many years^3^, ^4^ supports this hypothesis. Nevertheless, the best management after detection of ANCA in asymptomatic patients using MMI remains undefined.

Little information is available regarding the behavior of ANCA after MMI discontinuation.^6^ One study showed a reduction in ANCA after the withdrawal of ATD (PTU in most cases) in 98% of patients, but normalization was found in only 18%.^20^ On the other hand, another study demonstrated a negative ANCA test in 90% of patients 2 years after PTU discontinuation.^21^ The time necessary for antibody normalization and whether a difference exists between patients treated with PTU or MMI remain unknown. Our finding of a similar prevalence in patients under treatment versus those without treatment for at least 6 months (mean 3 years) suggests that a possible decline or negative ANCA test does not occur in the short term.

We recognize some limitations of the present study, which have already been addressed in the previous paragraphs. First, the sample was not large. However, we reiterate that this prospective clinical study was the first involving a Brazilian population. Second, in view of the limitation of our outpatient clinic that receives hyperthyroid patients already receiving ATD, we did not have a group not treated with MMI and we therefore used the data of previous comparative studies. Third, long-term follow-up is desirable since it could increase the frequency of ANCA detection, identify clinical vasculitis in patients with ANCA who continue under treatment, and show the course of antibodies after MMI discontinuation. Finally, ANCA were measured by indirect immunofluorescence (IFI). If positive, ELISA for MPO-ANCA and PR3-ANCA would follow.^7^ This step was not performed in the present study. However, we emphasize that specifically in cases with positive p-ANCA by IFI, observed in 15/17 of the present patients, there is usually agreement with MPO-ANCA 3+ or 2+.^7^ Even in cases with negative MPO-ANCA or 1+, this result may occur in treated, inactive or relapsing vasculitis,^7^ and can therefore not be considered irrelevant.

## Conclusion

This prospective clinical study of a Brazilian population shows a considerable frequency of ANCA in patients treated with MMI. However, since none of the ANCA-positive patients had clinical vasculitis, the clinical repercussion of this finding remains undefined and requires further studies.

## Ethical standards

The study was approved by the local Research Ethics Committee (CAAE: 72093917.6.0000.5138/no. 2.194.997) and the subjects gave written informed consent.

## Conflicts of interest

The authors declare no conflicts of interest.
